# MYB and bHLH transcription factor transgenes increase anthocyanin pigmentation in petunia and lisianthus plants, and the petunia phenotypes are strongly enhanced under field conditions

**DOI:** 10.3389/fpls.2014.00603

**Published:** 2014-11-05

**Authors:** Kathy E. Schwinn, Murray R. Boase, J. Marie Bradley, David H. Lewis, Simon C. Deroles, Cathie R. Martin, Kevin M. Davies

**Affiliations:** ^1^New Zealand Institute for Plant and Food Research Limited, Palmerston NorthNew Zealand; ^2^New Zealand Institute for Plant and Food Research Limited, WellingtonNew Zealand; ^3^John Innes Centre, Norwich Research Park, NorwichUK

**Keywords:** GMO, petunia, pigment, flavonoid, *Eustoma*, transgenic, field trial

## Abstract

*Petunia* line Mitchell [MP, *Petunia axillaris* × (*P. axillaris* × *P. hybrida*)] and *Eustoma grandiflorum* (lisianthus) plants were produced containing a transgene for over-expression of the R2R3-MYB transcription factor [TF; ROSEA1 (ROS1)] that up-regulates flavonoid biosynthesis in *Antirrhinum majus*. The petunia lines were also crossed with previously produced MP lines containing a *Zea mays* flavonoid-related basic helix-loop-helix TF transgene (LEAF COLOR, LC), which induces strong vegetative pigmentation when these *35S:LC* plants are exposed to high-light levels. *35S:ROS1* lisianthus transgenics had limited changes in anthocyanin pigmentation, specifically, precocious pigmentation of flower petals and increased pigmentation of sepals. RNA transcript levels for two anthocyanin biosynthetic genes, *chalcone synthase* and *anthocyanidin synthase*, were increased in the *35S:ROS1* lisianthus petals compared to those of control lines. With MP, the *35S:ROS1* calli showed novel red pigmentation in culture, but this was generally not seen in tissue culture plantlets regenerated from the calli or young plants transferred to soil in the greenhouse. Anthocyanin pigmentation was enhanced in the stems of mature *35S:ROS1* MP plants, but the MP white-flower phenotype was not complemented. Progeny from a *35S:ROS1* × *35S:LC* cross had novel pigmentation phenotypes that were not present in either parental line or MP. In particular, there was increased pigment in the petal throat region, and the anthers changed from yellow to purple pigmentation. An outdoor field trial was conducted with the *35S:ROS1*, *35S:LC*, *35S:ROS1* × *35S:LC* and control MP lines. Field conditions rapidly induced intense foliage pigmentation in *35S:LC* plants, a phenotype not observed in control MP or equivalent *35S:LC* plants maintained in a greenhouse. No difference in plant stature, seed germination, or plant survival was observed between transgenic and control plants.

## INTRODUCTION

Anthocyanins are a major group of pigments involved in a large range of plant functions. They are also key to the consumer appeal of many ornamental, fruit and vegetable products, and are of increasing interest to researchers for their possible dietary benefits to human health (Gould et al., 2008; Martin et al., 2011; Davies and Espley, 2013). Their biosynthesis, as part of the larger phenylpropanoid pathway, is well characterized at both the biochemical and molecular level (Grotewold, 2006). In addition to DNA sequences being available for most of the biosynthetic enzymes, sequences have also been identified from several species for proteins that regulate the biosynthetic gene transcription (Grotewold, 2006; Feller et al., 2011; Hichri et al., 2011; Davies et al., 2012). Of particular note are R2R3-MYB and basic helix-loop-helix (bHLH) type transcription factors (TFs) that form a complex with a WD-Repeat protein (the “MBW” complex) to activate the biosynthetic genes. This co-action of R2R3-MYB and bHLH proteins in regulating anthocyanin biosynthesis has been consistently found to occur in a range of monocot and eudicot species. Based on mutant analyses, in eudicots, the anthocyanin-related TFs are principally involved in activating a subset of the anthocyanin biosynthetic genes, the late biosynthetic genes (LBGs), with other R2R3MYBs being the principal regulators of the early biosynthetic genes (EBGs; Grotewold, 2006; Feller et al., 2011).

The availability of anthocyanin-related TF genes has allowed their use to modify the amount and spatial distribution of anthocyanins produced in transgenic plants and cell lines of several species (Schwinn and Davies, 2006; Hichri et al., 2011; Dixon et al., 2013). Indeed, TF genes have increasing become the focus of biotechnology approaches to altering plant metabolism, as they can coordinately regulate several biosynthetic genes, overcoming the need to identify a rate-limiting step or introduce multiple transgenes for biosynthetic enzymes. Despite the successes with anthocyanin TF transgenes, there are also cases where no phenotypic change has been induced in the transgenic plants (Bradley et al., 1999), or where the pigmented phenotype is only observed under stress conditions of high-light or cold (Goldsbrough et al., 1996; Ray et al., 2003; Albert et al., 2009; Rowan et al., 2009). This suggests environmental induced changes in the action of endogenous regulatory partners of the TF transgene product are required for effective activation of the anthocyanin pathway. Moreover, some flavonoid related R2R3-MYB transgenes have induced pleiotropic changes in the transgenic plants. For example, tomato plants over-expressing the grapevine VvMYB5a and VvMYB5b had not only modified flavonoid production but also dwarfism and changes in chlorophyll and β-carotene production (Mahjoub et al., 2009).

*Petunia* is an established model species for understanding the regulation of anthocyanin production and its modification in transgenic plants. *Petunia* has specific vegetative and floral anthocyanin pigmentation patterns that are determined by the expression patterns of individual R2R3-MYB and bHLH activators (Quattrocchio et al., 1998, 1999; Albert et al., 2011, 2014) and R3-MYB and R2R3-MYBs with a repressive action on anthocyanin biosynthesis (Albert et al., 2014). Over-expression of the maize bHLH *Leaf color* (*35S:LC*) in the petunia line Mitchell (MP, which contains a mutation in the main petal R2R3-MYB activator, *an2*) enhanced light-induced vegetative and floral pigmentation phenotypes (Albert et al., 2011). In particular high anthocyanin levels, producing dark colored leaves, were generated in *35S:LC* MP plants grown under high-light conditions in growth chambers. However, over-expression of two endogenous R2R3-MYB activators, DEEP PURPLE (DPL) and PURPLE HAZE (PHZ), generated MP lines that accumulated high levels of anthocyanins in their leaves under standard greenhouse conditions, without the high-light requirement of *35S:LC* plants. Albert et al. (2014) showed that the expression of the *LC* transgene did not change with light conditions, but transcripts for DPL and PHZ increased in abundance while those for the R2R3-MYB repressor MYB27 decreased upon high-light treatment. These TF transgenic lines have proven very useful in elucidating the interactions of the MBW components in the regulation of anthocyanin biosynthesis (Albert et al., 2014).

In *Antirrhinum majus* (antirrhinum), the *Rosea1* (*ROS1*), *Rosea2,* and *Venosa* genes encode R2R3-MYB TFs that regulate anthocyanin pigmentation of the flowers, in conjunction with the bHLH factors DELILA and MUTABILIS (Goodrich et al., 1992; Schwinn et al., 2006; Shang et al., 2011). *ROS1* is required for wild-type levels of pigmentation in the flowers and vegetative parts, while *Rosea2* and *Venosa* determine specific pigmentation patterns, for example venation (Schwinn et al., 2006; Shang et al., 2011).

In the study reported here the effect of introducing a transgene for production of ROS1 into the heterologous ornamental species petunia and lisianthus (*Eustoma grandiflorum*) has been examined. These species belong to two Asterid orders, the Solanales (petunia) and Gentianales (lisianthus), that contain many economically important ornamental species and that are generally placed next to each other in phylogenetic trees. While many studies have been conducted on the effects of anthocyanin-related TF transgenes in Solanales species, studies are lacking for any Gentianales species. The plants studied include R2R3-MYB/bHLH double transgenic petunias, generated by crossing the *35S:ROS1* lines with the *35S:LC* lines characterized by Albert et al. (2009, 2011, 2014). The influence of the environment on the phenotypes of the *35S:LC*, *35S:ROS1*, and *35S:LC*/*35S:ROS1* petunia lines was assessed by comparison of plants in an outdoor field trial to those in standard greenhouse conditions. As flavonoids have multiple roles in plants, including influences on auxin transport (Peer et al., 2011; Cheynier et al., 2013), changes in the pathway activity may alter general plant performance, as observed in tomato (Mahjoub et al., 2009). Moreover, anthocyanin-based vegetative pigmentation has been postulated to help to improve the general stress tolerance of leaves (Gould et al., 2008), and petunia plants with increased anthocyanin levels in leaves showed increased light saturation and light compensation points without reducing the maximal photosynthetic assimilation rate (Albert et al., 2009). Therefore, the transgenics were assessed not only for the changes in anthocyanin production, but also for seed germination rate, plant stature, and plant survival under field conditions.

## MATERIALS AND METHODS

### PLANT MATERIAL AND GREENHOUSE CONDITIONS

The ‘Mitchell’ (W115) petunia line (MP) was obtained from Professor Richard Gardner at the University of Auckland, New Zealand, and is *Petunia axillaris* × (*P. axillaris* × *P. hybrida* cv. ‘Rose of Heaven’; Cornu and Farcy, 1981). The *35S:LC* MP transgenic line is as described in Bradley et al. (1998), and which was further characterized in Albert et al. (2009, 2011, 2014). The origin of the purple-flowered lisianthus cv. 54 is described in Davies et al. (1993), and further characterized in Nielsen et al. (2002). Flower development in lisianthus cv. 54 is divided into six stages: stage 1 – bud less than 1.5 cm in length; stage 2 – bud length 1.5–2.5 cm; stage 3 – bud length greater than 2.5 cm, petals tightly furled; stage 4 – petals starting to unfurl, anthocyanin first present in the petal tips; stage 5 – flowers opening, petals colored throughout; stage 6 – flowers fully open with maximum anthocyanin levels (Davies et al., 1993). Plants were grown under standard greenhouse conditions in Palmerston North, New Zealand. The greenhouse was heated at 15∘C and vented at 25∘C, without supplementary lighting. Plant color phenotypes were assed using a Minolta CR-200 tri-stimulus colorimeter with a D65 light (Lewis et al., 1998). The colorimeter readings were compared by one-way analysis of variance (ANOVA), calculated with Genstat software. Least significant difference (LSD) values are presented to allow comparison of calculated means.

### BINARY VECTOR CONSTRUCTION

The full-length *ROS1* cDNA was ligated into the *Kpn*I site of pART7 (Gleave, 1992) in the ‘sense’ orientation between the 35SCaMV promoter and the octopine synthase (OCS) terminator sequence, to form pLN81. The *35S:ROS1:OCS* cassette was taken as a *Not*I fragment then ligated into the *Not*I site of pART27 (Gleave, 1992), which contains a kanamycin selectable marker, resulting in the binary vector pLN83. DNA sequencing confirmed the integrity of the T-DNA region.

### *Agrobacterium tumefaciens* MEDIATED TRANSFORMATION OF MP AND LISIANTHUS

Generation of transgenic MP plants used the method of Deroles and Gardner (1988), based on co-cultivation of leaf disks with the disarmed *Agrobacterium tumefaciens* strain LBA4404 (Hoekema et al., 1983). Generation of transgenic lisianthus plants was by the method of Ledger et al. (1997), based on co-cultivation of leaf disks with the virulent *A. tumefaciens* strain A722, a C58 derivative cured of pTiC58 and containing the octopine pTiA6NC (Garfinkel and Nester, 1980). The leaf disks for both species were from young leaf tissue from clonal plants that were greenhouse grown.

### FIELD TRIAL REGULATORY PROCESS, CONDITIONS AND LOCATION

**FIGURE 1:**
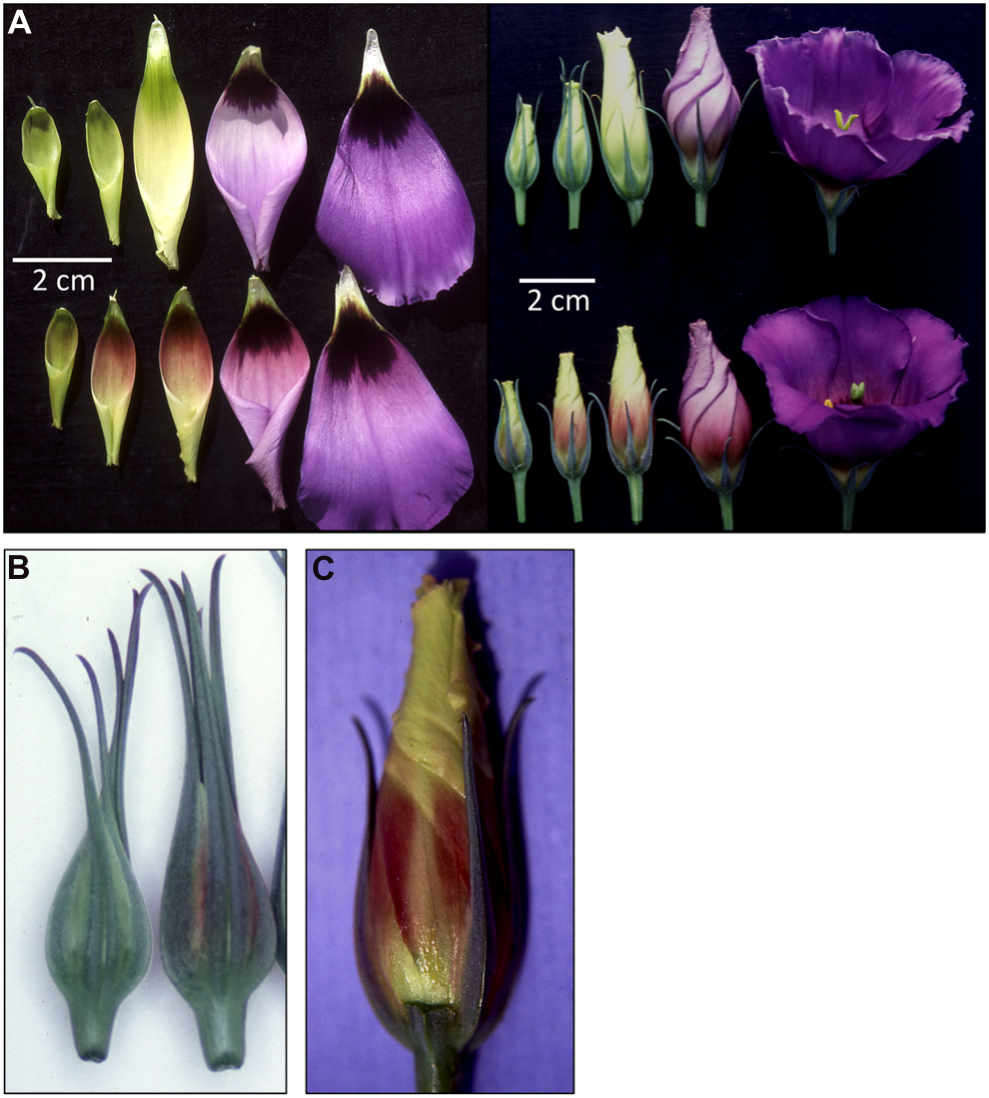
**Phenotypes of *35S:ROS1* lisianthus transgenics. (A)** Whole flowers and isolated petals of non-transgenic lisianthus cv. 54 (top) and *35S:ROS1* transgenic 54-14 (bottom) over five stages of development. **(B)** Close up of buds of one non-transgenic plant (left) and line 54-14 (right), showing the increased pigmentation that occurs in the sepals of the transgenic. **(C)** A flower bud of line 54-14 with a sepal removed to show the reduced level of petal pigmentation underneath the sepals.

Approval to conduct a field trial with six transgenic lines and two non-transgenic control lines of MP (to a total of 160 plants) was granted under Section 39(1b) of the New Zealand Government (1996) by the GMO Special Committee of the New Zealand Environmental Risk Management Authority. The trial approval required that no open flowers were allowed to form on the transgenic plants, precluding assessment of pollen transfer from transgenic to neighboring non-transgenic lines and the study of plant lines modified for altered flower color. Thus, the plant lines included in the field trial were those modified for vegetative phenotypes and an appropriate control line (**Table 1**; **Figure 1**). The plants were grown from seed, germinated under standard commercial conditions in either open seed trays or cell trays. Once seedlings were well established they were grown on in pots prior to field planting. The field test was located near Palmerston North, New Zealand, and was planted on November 22nd 1999 and grown for 3 months until destruction on February 22nd 2000.

**Table 1:** Plant lines used in the field trial.

Plant line	Line description	T -DNA copy number
1	Seedlings from a self-pollinated plant of 38/MP/118^1^ (*35S:LC* × )	1 or 2
2	Seedlings from 38/MP/118 × MP (*35S:LC* × MP back-cross)	1 or 2
3 and 4	Seedlings of 38/MP/118 × 83/MP/231 (*35S:LC* x *35S:ROS1*)	ND
5	Seedlings from a self-pollinated plant of 83/MP/231 (*35S:ROS1* × )	ND
6	Seedlings of non-transgenic MP	–

*^1^see Bradley et al. (1998).*

*ND, not determined.*

### RNA AND DNA EXTRACTION AND ANALYSIS

RNA was isolated using either the RNeasy Mini Kit (Bio-strategy, Auckland, New Zealand) or TRIzol Reagent protocol (Life Technologies, Auckland, New Zealand). DNA was isolated using the Boehringer Plant DNA Isolation Kit (Roche, Auckland, New Zealand). Northern RNA analysis was as described in Deroles et al. (1998). Final wash stringency was 65∘C and 0.1 × SSC/0.1% SDS. Probes were cDNA inserts for *ROS1* (antirrhinum), anthocyanidin synthase (*ANS*) and chalcone synthase-A (*CHS*) from *P. hybrida*, and CHS and ANS from lisianthus. RNA was quantified using UV-spectrophotometry and equivalent RNA loadings confirmed using ethidium bromide staining of RNA in the gels and hybridization of the northern membranes with a cDNA for a 25/26S rRNA from *Asparagus officinalis*. For Southern analysis, 30 μg genomic DNA was digested for several hours at 37∘C in a 50 μL volume with either *Asp*718 (which releases the full-length *ROS1* cDNA) to check for intact T-DNA copies or *Eco*RI (which generates T-DNA junction fragments) to assay T-DNA copy number. Hybridization was as in Deroles et al. (1998), with a final wash stringency was 65∘C and 0.1 × SSC/0.1% SDS. The analysis included lambda *Hind*III DNA for size markers and the *Asp*718 full-length *ROS1* cDNA fragment as a positive control.

## RESULTS

### PHENOTYPES OF KANAMYCIN-RESISTANT *35S:ROS1* LISIANTHUS CALLI, SHOOTS AND PLANTS IN CULTURE

Approximately 160 independent shoots were harvested from lisianthus leaf pieces inoculated with *A. tumefaciens* containing the *35S:ROS1* vector. The putative transgenic tissue did not show any differing phenotypes in culture than culture lines derived from transformation with non-anthocyanin related transgenes (data not shown).

### PHENOTYPES OF MATURE *35S:ROS1* LISIANTHUS TRANSGENICS

Fourteen independent kanamycin-resistant lisianthus lines of cv. 54 were transferred from culture into the greenhouse and grown to maturity. Three lines had no phenotype changes compared to non-transgenic lines, but nine lines exhibited temporal, spatial and quantitative changes in anthocyanin pigmentation. In cv. 54, petal anthocyanin coloration begins at stage 4 when the petals are nearly fully expanded and starting to unfurl (Davies et al., 1993). It begins in the tips of the petals, and increases down throughout the inner epidermis and the outer epidermis of the rest of the petal during flower development (**Figure 1A**). The petals also have a dark ‘eye’ (**Figure 1A**), which commences pigmentation at the same stage. In some of the *35S:ROS1* transgenics petal anthocyanin pigmentation started at stage 2, including in the eye, and was first apparent in the lower part of the petal rather than at the tips (**Figures 1A,B**). Petal tissue that was covered by the sepals had less visible pigmentation than the petal tissue fully exposed to the light (**Figure 1C**). Transgenic line 54-14 showed the strongest phenotype, followed by 54-111, while other lines, such as 54-11 and 54-15, had much weaker phenotypes. Examples of both types were included in the further molecular analysis.

The pigmentation that developed in the early bud stages of the *35S:ROS1* transgenics was redder than the pigmentation that occurred later in development or the pigmentation that developed in control flowers (**Figure 1A**). As buds matured from stage 4 to 6, further pigmentation appeared to develop normally, and in mature flowers, color and intensity of pigmentation was similar to flowers from untransformed plants (**Figure 1A**). Changes also occurred in sepal pigmentation. Normally in cv. 54, anthocyanins are readily visible only in the sepal tips, however, in transgenic with altered petal pigmentation anthocyanins were also clearly visible in the sepal bases (**Figure 1B**).

### SOUTHERN DNA ANALYSIS OF *35S:ROS1* LISIANTHUS TRANSGENICS

**FIGURE 2:**
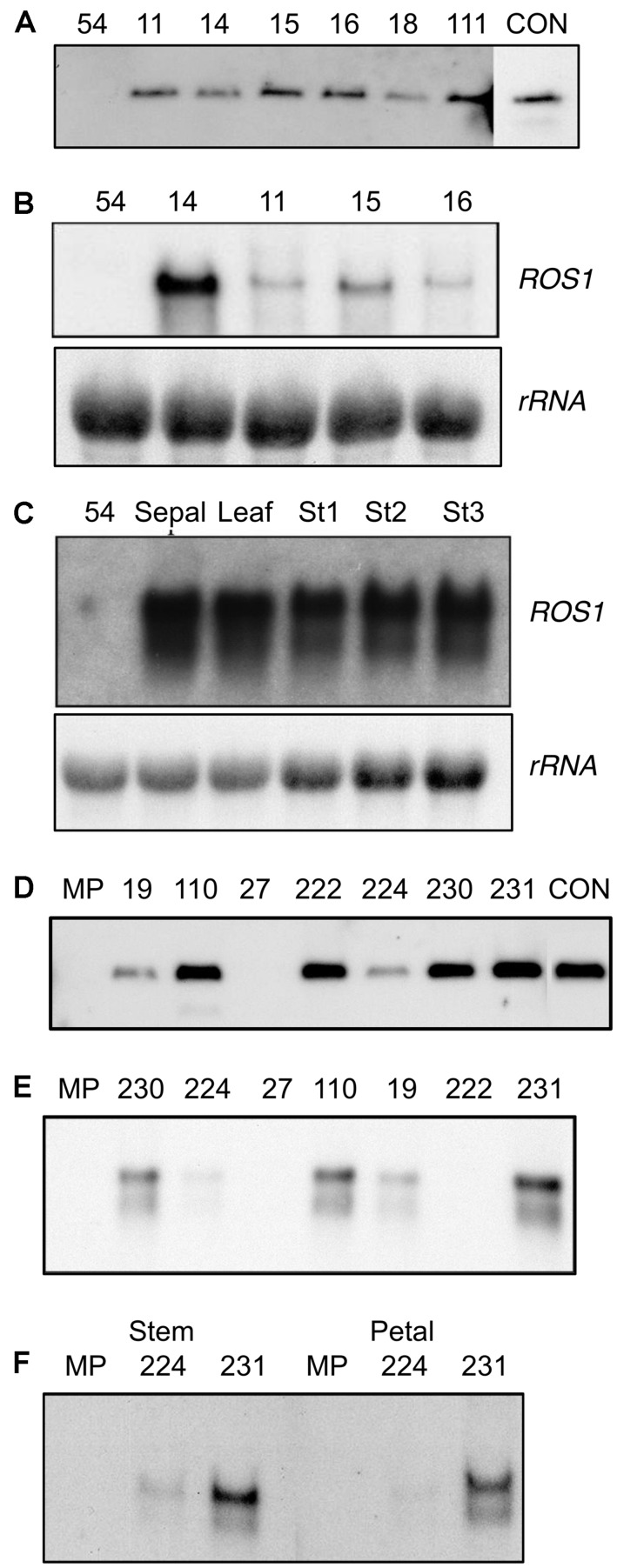
**Analysis of *35S:ROS1* plants for the presence and expression of the transgene. (A)** Southern hybridization of *35S:ROS1* lisianthus plants using radiolabelled ROS1 cDNA as the probe. Thirty μg of genomic DNA was digested with the restriction enzyme Asp718 to release the ROS1 fragment from the T-DNA. The control (CON) lane contained pLN81-derived DNA and shows the expected fragment from an Asp718 digest. The lane labeled ‘54’ contains genomic DNA from a non-transgenic lisianthus cv. 54 plant. **(B)** Northern hybridization analysis of ROS1 transcript abundance in petal RNA of a non-transgenic control plant (54) and four lines of *35S:ROS1* lisianthus (14, 11, 15, 16). **(C)** Northern hybridization analysis of ROS1 transcript abundance in sepal, leaf, and three petal stages (Stage 1,3 and 5) of *35S:ROS1* lisianthus line 54-14 and petal RNA of a non-transgenic control plant (54). In both **(B)** and **(C)** 20 μg of total RNA was hybridized with radiolabelled ROS1 cDNA or an rRNA cDNA (for an estimation of RNA loading) as the probe. **(D)** Southern hybridization of *35S:ROS1* MP petunia plants using radiolabelled ROS1 cDNA as the probe. Thirty μg of genomic DNA was digested with the restriction enzyme Asp718 to release the ROS1 fragment from the T-DNA. The control (CON) lane contained pLN81-derived DNA and shows the expected fragment from an Asp718 digest. The lane labeled ‘MP’ contains genomic DNA from a non-transgenic MP plant. **(E)** Northern hybridization analysis of ROS1 transcript abundance in total RNA (10 μg) from petals of seven *35S:ROS1* MP plants and a non-transgenic control (MP). RNA was hybridized with radiolabelled ROS1 cDNA as the probe. **(F)** Northern hybridization analysis of ROS1 transcript abundance in total RNA from stem (25 μg) or petal (30 μg) RNA from *35S:ROS1* lines MP224 and MP231 and a non-transgenic control (MP).

Southern DNA analysis detected *ROS1*-hybridizing fragments of the size expected for the *35S:ROS1* T-DNA in all the kanamycin-resistant lisianthus lines transferred to the greenhouse (**Figure 2A**).

### ROS1 EXPRESSION IN *35S:ROS1* LISIANTHUS TRANSGENICS

Northern analysis using the *ROS1* cDNA as a probe showed transcript of the expected size in RNA from petal tissue of all the *35S:ROS1* lisianthus lines (**Figure 2B**). However, *ROS1* transcript abundance was highest in RNA from lines displaying the pigmentation phenotype, such as 54-14, and much lower in those with weak phenotypes, such as 54-11 and 54-15 (**Figure 2B**). *ROS1* transcript abundance was similar in RNA samples from stages 1, 3, or 5 and was also detected in leaf RNA samples (**Figure 2C**).

### ANTHOCYANIN BIOSYNTHETIC GENE EXPRESSION IN *35S:ROS1* LISIANTHUS TRANSGENICS

**FIGURE 3:**
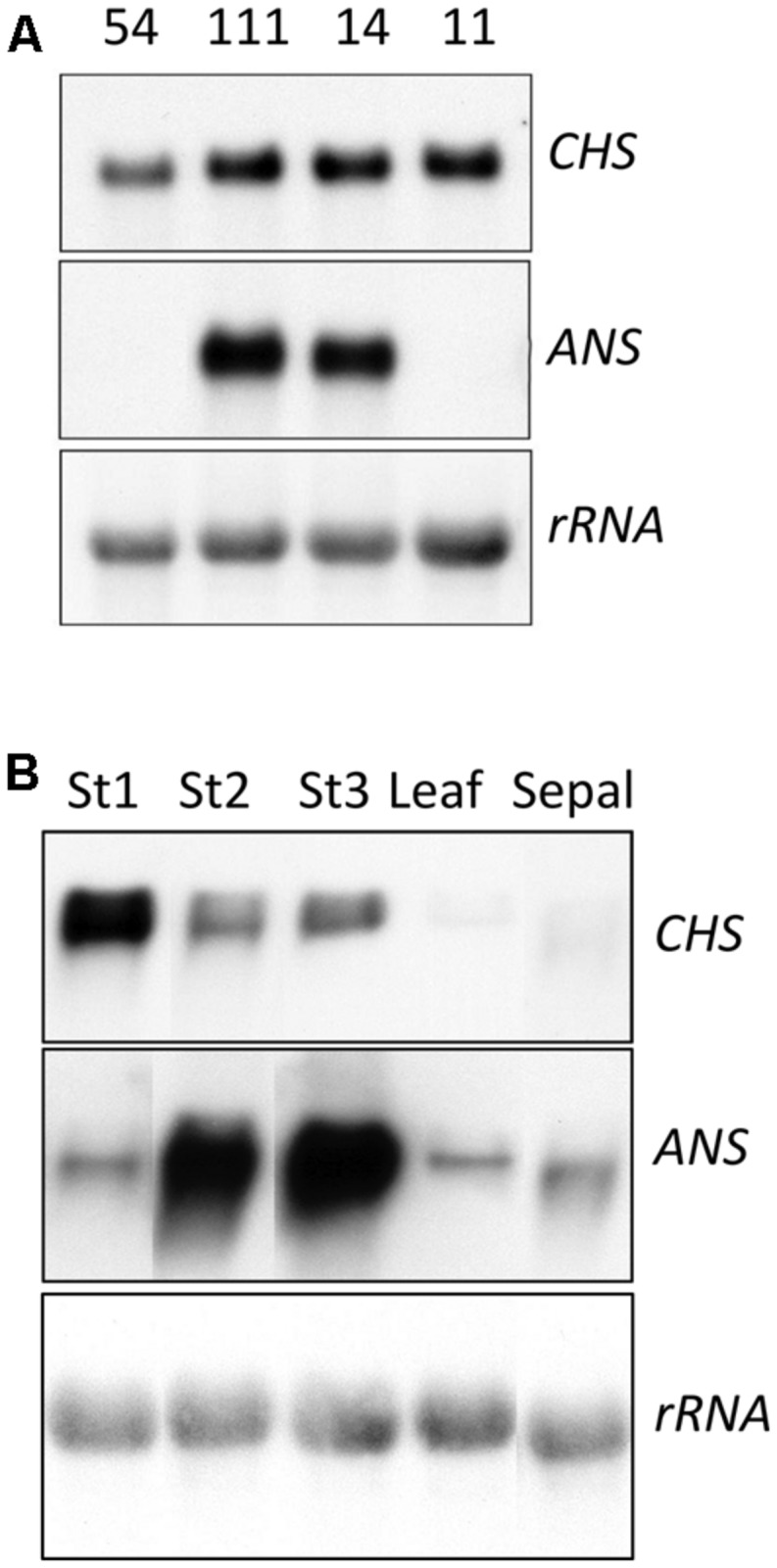
**Analysis of transcript abundance for the anthocyanin biosynthesis genes CHS and ANS in *35S:ROS1* and non-transgenic lisianthus plants. (A)** RNA samples (10 μg total RNA) from Stage 3 petals of three *35S:ROS1* transgenic lines (54-111, 54-14, and 54-11) and a non-transgenic (54) line. **(B)** RNA samples (20 μg of total RNA) from three petal stages (Stage 1,3, and 5), sepal, leaf of transgenic line 54-14. Northern RNA hybridisation was conducted against radiolabelled CHS, ANS and rRNA cDNA probes.

Transcript levels for two representative anthocyanin biosynthetic genes, the EBG *CHS* and the LBG *ANS*, were analyzed in RNA from stage three petal tissue of two lines with a strong phenotype and high expression of *ROS1* (54-14 and 54-111), one line with no phenotype and low *ROS1* expression (54-11), and one non-transgenic control (**Figure 3A**). Petal *CHS* transcript levels were elevated in all three transgenics compared to the controls. *ANS* transcript was not detected in the control lines or 54-11 but was readily detected in lines 54-14 and 54-111. Line 54-14, which has the strongest anthocyanin phenotype, had the highest *ANS* transcript levels. *CHS* and *ANS* transcript levels in line 54-14 showed marked variation over the period of petal development (**Figure 3B**). *CHS* transcript was most abundant in stage one RNA samples, while *ANS* transcript abundance increased from stage one through to stage five.

### PHENOTYPES OF *IN VITRO* KANAMYCIN-RESISTANT *35S:ROS1* MP TISSUE

**FIGURE 4:**
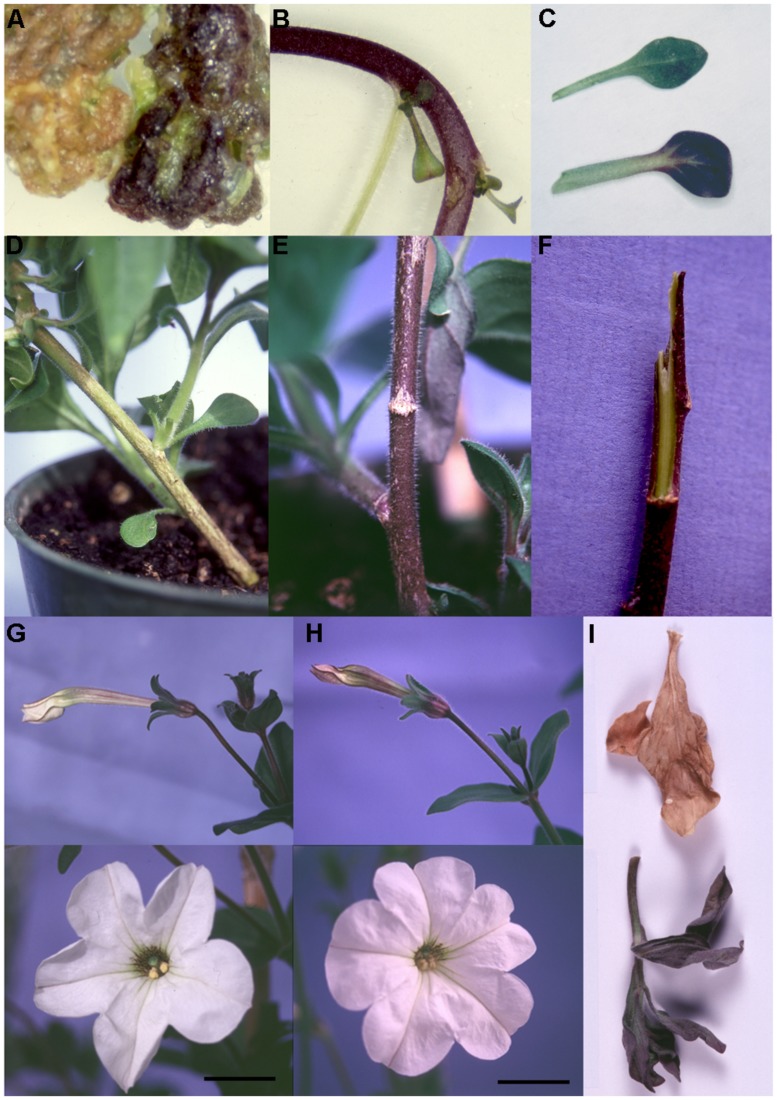
**Phenotypes of *35S:ROS1* MP transgenics. (A)** Comparison of non-transgenic (left) and kanamycin-resistant *35S:ROS1* (right) callus in in vitro culture. **(B)** Close up of the stem tissue of a kanamycin-resistant *35S:ROS1* plantlet in culture. **(C)** Leaves from non-transgenic (top) and *35S:ROS1* (bottom) plants. **(D)** Close up of the base of a non-transgenic. **(E)** Close up of the base of *35S:ROS1* transgenic MP231, showing the pigmented stem tissue. **(F)** A stem from *35S:ROS1* transgenic MP231 with the outer cell layers removed, showing the lack of pigmentation in the central stem tissues. **(G)** A bud (top) and flower (bottom, scale bar = 2 cm) of a non-transgenic MP plant. **(H)** A bud (top) and flower (bottom, scale bar = 2 cm) from *35S:ROS1* transgenic MP224. **(I)** Examples of senesced leaves, of a similar developmental age, from a non-transgenic MP (top) and transgenic line MP110 (bottom).

Approximately 200 shoots or calli representing independently transformed loci were dissected from MP leaf disks inoculated with *A. tumefaciens* containing a *35S:ROS1* vector, and allowed to proliferate. The first phenotype observed was purple pigmentation of some calli (**Figure 4A**), a phenotype not observed in calli derived from untransformed MP or MP transformed with non-anthocyanin related transgenes (**Figure 4A** and data not shown). Pigmentation of calli was restricted to the outer layer(s) of cells, and was predominantly on the top portions of the calli, which were not embedded in the medium. Both un-pigmented and pigmented shoots arose from the callus, and pigmentation was also observed sporadically in leaves and stems of some plantlets (**Figures 4B,C**).

### PHENOTYPES OF MATURE *35S:ROS1* MP TRANSGENICS

Fifty-eight independent kanamycin-resistant *35S:ROS1* MP lines were transferred to the greenhouse and grown to flowering. No pigmentation phenotype was observed when the plants were young, but anthocyanin pigmentation was enhanced in the stems of mature plants compared to non-transgenic MP (**Figures 4D–F**). However, none of the lines showed complementation of the MP white-flower phenotype (**Figures 4G,H**). The increase in pigmentation was most notable in the main stem at the base of the plant, but also occurred in the lower regions of branching stems toward the base of the plant and in the senescing leaves (**Figure 4I**).

Detailed analysis of the *35S:ROS1* MP focused on lines MP19, MP110, MP27, MP222, MP224, MP230, and MP231. This group displayed varying levels of stem pigmentation; lines MP110, MP19, and MP231 had strong pigmentation, MP230 had moderate pigmentation, and MP27, MP224, and MP222 had weak pigmentation. Line MP27, which was thought to lack an intact *35S:ROS1* transgene had the least pigmentation.

### SOUTHERN DNA ANALYSIS OF *35S:ROS1* MP TRANSGENICS

Southern DNA analysis was used to determine whether the transformants contained intact copies of the *35S:ROS1* transgene. Seven independent transgenic lines from the 58 that were transferred from culture into the greenhouse were selected for Southern analysis. Six of the seven lines showed bands on a Southern corresponding to intact copies of the *35S:ROS1* transgene (**Figure 2D**), but no transgene was detected in line MP27.

### ROS1 EXPRESSION IN *35S:ROS1* MP TRANSGENICS

Northern analysis using the *ROS1* cDNA as a probe showed transcript of the expected size in RNA from petal tissue of lines MP231, MP110, MP230 19 and 224 (**Figure 2E**). Two transcripts were found, with the larger of the two main transcripts likely to be due to alternative transcription termination. *ROS1* transcript was detected in both pigmented (stem) and un-pigmented (petal) tissue (**Figure 2F**).

### ANTHOCYANIN BIOSYNTHETIC GENE EXPRESSION IN *35S:ROS1* MP TRANSGENICS

**FIGURE 5:**
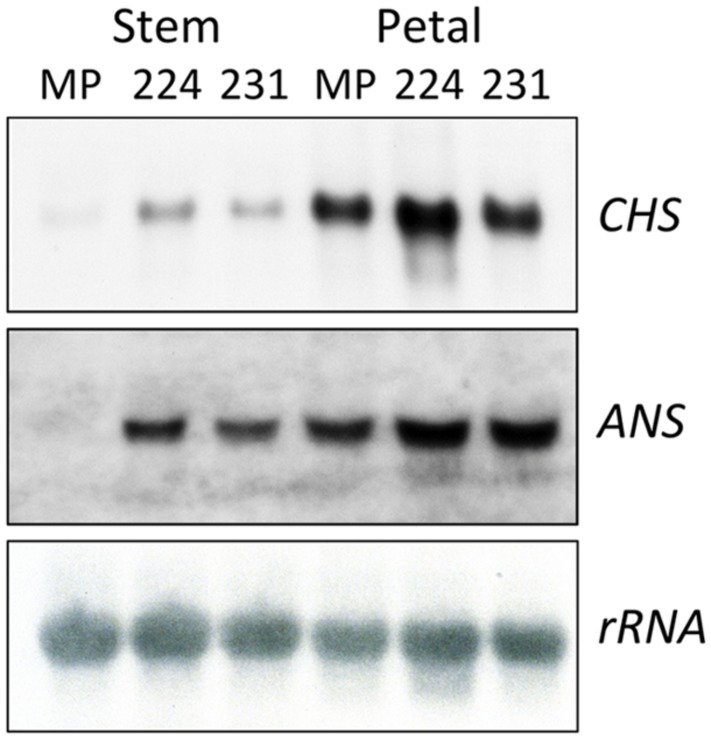
**Analysis of transcript abundance for the anthocyanin biosynthesis genes CHS and ANS in *35S:ROS1* (MP224 and MP231) and non-transgenic (MP) petunia lines**. Twenty (CHS) or 50 (ANS) μg of total RNA from stems and petals of transgenic lines was hybridized in northern RNA analysis with radiolabelled CHS, ANS or rRNA cDNA probes.

Induced changes in transcript abundance for CHS and ANS were examined using RNA from both pigmented (stem) and un-pigmented (petal) tissue of two *35S:ROS1* transgenic lines, and from non-transgenic MP (**Figure 5**). Transcript levels for ANS and CHS in *35S:ROS1* stem RNA samples were higher than for non-transgenics, with ANS showing a more marked increase than CHS (**Figure 5**). In RNA from un-pigmented petal tissue, *CHS* transcript levels showed no marked change and *ANS* transcript levels were only weakly elevated.

### PHENOTYPES OF MP TRANSGENICS CONTAINING BOTH MYB AND bHLH TRANSGENES

**FIGURE 6:**
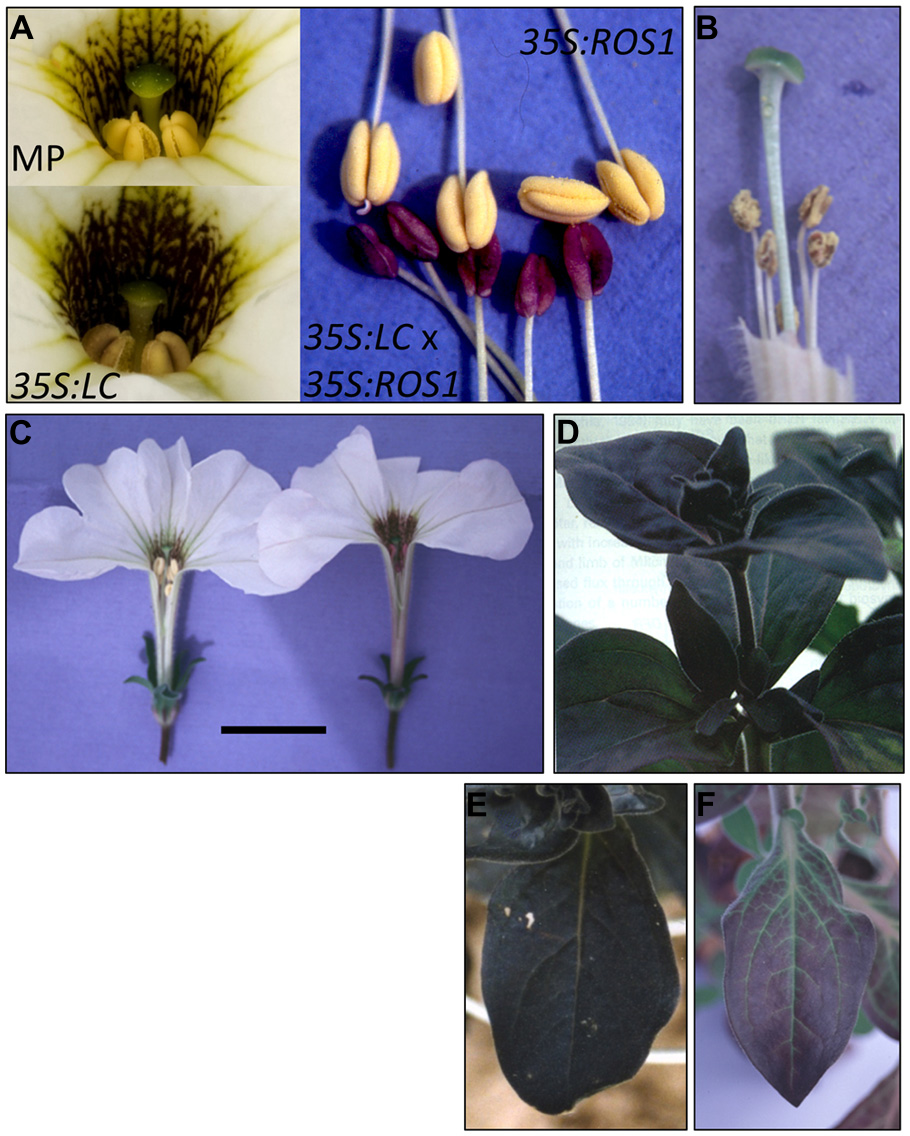
**Phenotypes of progeny of self-pollination of *35S:ROS1* Mitchell petunia and crosses between *35S:ROS1* and *35S:LC* MP transgenics. (A)** Anthers from MP (top left), a *35S:LC* plant (bottom left), a *35S:ROS1* plant (top right), and a *35S:ROS1* x *35S:LC* plant (bottom right). **(B)** Dehisced anthers from a *35S:ROS1* x *35S:LC* plant, showing the lack of pigment in pollen. **(C)** Comparison of flowers from a non-transgenic plant (left) and a *35S:ROS1* x *35S:LC* plant (right), showing the increased pigmentation of the throat region and the pigmented anthers of the double transgenic. Scale bar represents 2 cm. **(D)** and **(E)** Leaves of a *35S:LC* plant. **(F)** A leaf from a *35S:ROS1* x *35S:LC* plant.

Individuals of two primary transformants with strong stem pigmentation (MP110 and/or MP231) were self-pollinated or crossed to MP lines containing a *35S:LC* transgene (Bradley et al., 1998; Albert et al., 2009, 2011, 2014). Some progeny from selfed *35S:ROS1* or *35S:ROS1* × *35S:LC* showed possible enhanced stem pigmentation compared to the primary transformants, but as the plants were not grown under controlled environment conditions this was not considered as definitive. However, clear novel phenotypes did occur in the progeny from the *35S:ROS1* × *35S:LC* cross that were not present in either parental line or control MP. In particular, there was increased pigment in the petal throat (**Figure 6C**), and the anthers, which are yellow in MP and *35S:LC* lines, had purple pigmentation (**Figure 6A**). The pollen were not pigmented (**Figure 6B**). There were also variations in the leaf pigmentation phenotype normally observed for the *35S:LC* parental line, most notably an absence of pigmentation from the veins of the leaf blade in some progeny (compare **Figures 6D,E** to **Figure 6F**).

### COMPARISON OF MP TRANSGENICS GROWN IN OUTDOOR OR GREENHOUSE CONDITIONS

As environmental conditions have been shown to influence pigmentation phenotypes of plants containing anthocyanin-related TF transgenes for various species, permission was obtained to grow the MP transgenics in the field in the summer season in New Zealand. This also allowed the affect of the TFs on general plant performance to be assessed outside of the artificial conditions of pot-grown plants in a greenhouse. As field trial conditions required that the plants were not allowed to release pollen, only the vegetative phenotypes were recorded in the field.

A minimum of 20 plants each for *35S:ROS1*, *35S:LC* and *35S:ROS1* × *35S:LC* lines (**Table 1**) were grown in the field, along with the control MP line. All plants were germinated from seed in the greenhouse at the same time, in both cell trays and undivided seed trays. Germination frequencies were similar between transgenic and non-transgenic plants, with uniformly high rates of germination (**Table 2**). When the young plants were transferred to the field some of the same batch of plants were retained in the greenhouse and potted up into standard 150 mm pots. Survival rates over the 3 months of field growth were ≥ 75% for all lines, except for 55% for seedlings from a *35S:LC* × *MP* back-cross (line 2).

**Table 2:** Germination frequencies per plant line, assessed 14 days after sowing seed, and percentage of plants surviving at the end of the 3 month field trial.

Plant line	Total seedlings	Germination (%)	Plant survival
1 = *35S:LC*×	94/100	94	20/25 (75%)
2 = *35S:LC* × MP	94/101	93	20/29 (55%)
3 = *35S:LC* × *35S:ROS1*	93/100	93	20/24 (80%)
4 = *35S:LC* × *35S:ROS1*	101/102	99	20/23 (85%)
5 = *35S:ROS1*×	101/103	98	20/23 (85%)
6 = MP control	98/100	98	20/25 (75%)

**FIGURE 7:**
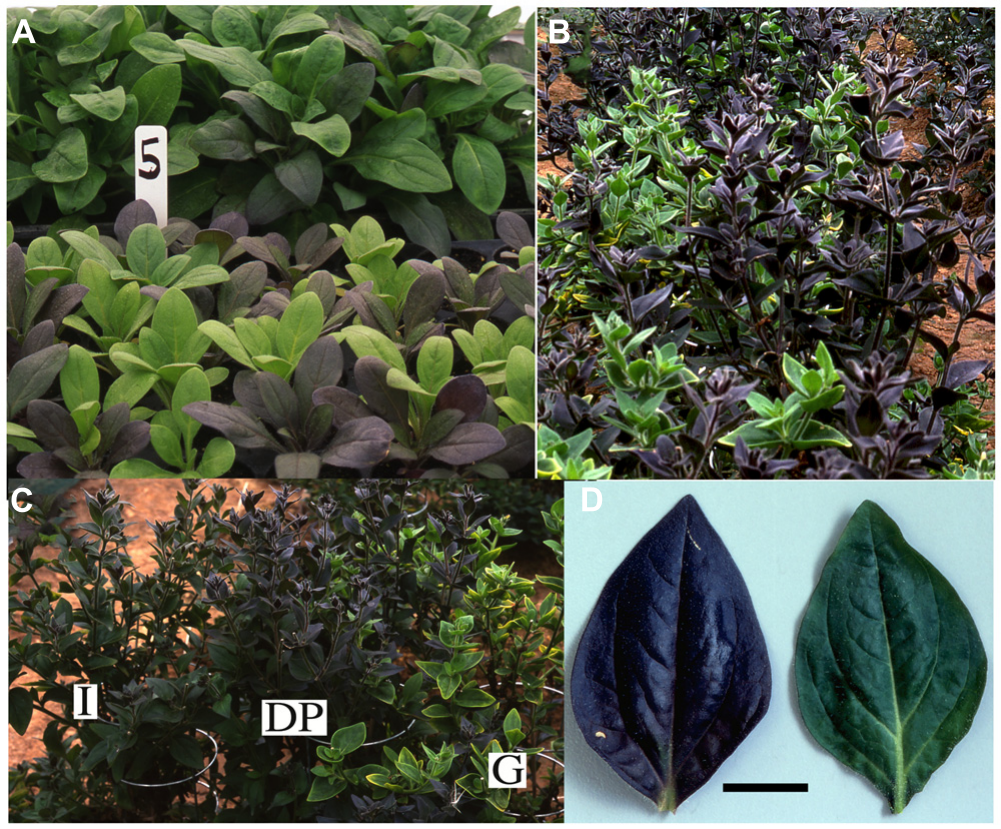
**Phenotypes of *35S:LC* Mitchell petunia lines grown in the greenhouse or the field. (A)** Seedlings in the greenhouse in individual cells in a cell tray (front) or in an open seed tray (rear). Note that the colored-foliage phenotype is more pronounced in the cell tray. **(B, C)** Mature plants in the field, showing dark purple (DP), intermediate (I) and green (G) phenotypes. **(D)** Leaves from plants of the same age planted in the field (left) or retained in the greenhouse (right). Scale bar represents 2 cm.

The color of the plant lines was assessed visually (**Table 3**), by comparison of tri-stimulus colorimeter readings (**Table 4**), and recorded photographically. A colored vegetative phenotype was apparent in the greenhouse in some of the seedlings containing a *35S:LC* transgene, particularly among those plants germinated in cell trays (**Figure 7**). This was consistent across all cell trays. However, this purple coloration decreased markedly after growth in individual pots prior to field planting. Once transferred to the field, individual plants containing the *35S:LC* transgene showed greatly increased anthocyanin pigmentation in leaf tissue, within 4 days of planting. Three distinct color phenotypes were present; green-leaved plants, purple-leaved and an intermediate, pale purple phenotype (**Figures 7B,C**; **Table 4**). The colored foliage phenotype was only observed with *35S:LC* plants, although weak stem pigmentation was observed with all plant lines toward the end of the trial period. The full purple color was clearly distinguishable using LCH tri-stimulus colorimeter readings, with a hue angle in the purple sector of the color wheel and reduced Lightness and Chroma value compared to the other phenotypes (**Tables 4** and **5**). This full purple phenotype was only found in line 1 (seedlings from a *35S:LC* self-cross), and only in field-grown plants. The *35S:LC* transgenic plants retained under greenhouse conditions only developed weak foliage pigmentation (**Figure 7D**; **Table 5**). Line 1 gave all three phenotypes in the field while line 2 (*35S:LC* × *MP*) produced only plants with the intermediate phenotype. To assess the colored anther phenotype of the *35S:ROS1* × *35S:LC* seedlings, flower buds were collected and allowed to develop in a containment laboratory. All flowers from non-transgenic, *35S:LC*, and *35S:ROS1* lines had yellow anthers. Twenty-one out of 33 flowers (64%) from the *35S:ROS1* × *35S:LC* seedlings had purple anthers.

**Table 3:** Frequency of plant color phenotypes per Mitchell petunia line at completion of the field trial.

Plant line	Number of plants	Green	Purple	Intermediate
1 = *35S:LC*×	15	5	4	6
2 = *35S:LC* × MP	11	0	0	11
3 = *35S:LC* × *35S:ROS1*	16	3	0	13
4 = *35S:LC* × *35S:ROS1*	17	2	0	15
5 = *35S:ROS1*×	17	17	0	0
6 = MP control	15	15	0	0

**Table 4:** Tri-stimulus colorimeter readings of leaves from plants of transgenic Mitchell petunia Line 2 (*35S:LC* × MP) exhibiting three color phenotypes.

	Purple	Intermediate	Green	LSD
**Lightness**	27.07	32.42	38.29	5.76
**Chroma**	0.81	8.85	22.19	14.2
**Hue**	352.75	120.55	124.75	79.80

*Lightness represents the proportion of total incident light that is reflected, Chroma is a measure of color intensity (in relative intensity units), and Hue angle relates color to a position on a color circle or wheel, with red-purple at 0∘, yellow at 90∘, blue-green at 180∘, and blue at 270∘. Multiple readings were taken from two independent plants for each data point, and the data presented are mean values. The LSD values were calculated at the 5% significance level, df = 3.*

**Table 5:** Tri-stimulus colorimeter readings of leaves from plants of Mitchell petunia (MP) or transgenic Line 1 (*35S:LC* x) grown in a greenhouse or the field.

Plant	Line 1 GH	Line 1 FD	MP GH	MP FD	LSD
**Phenotype**	Green	Purple	Green	Green	
**Lightness**	35.06	28.30	36.90	38.48	1.76
**Chroma**	16.50	1.32	20.31	18.28	3.66
**Hue**	132.70	314.50	131.20	128.30	38.90

*Plants of the same age were either transplanted into an open field (FD) or retained in the greenhouse (GH). Readings were taken from five independent plants for each data point, and the data presented are mean values. The LSD values were calculated at the 5% significance level, df = 16.*

**FIGURE 8:**
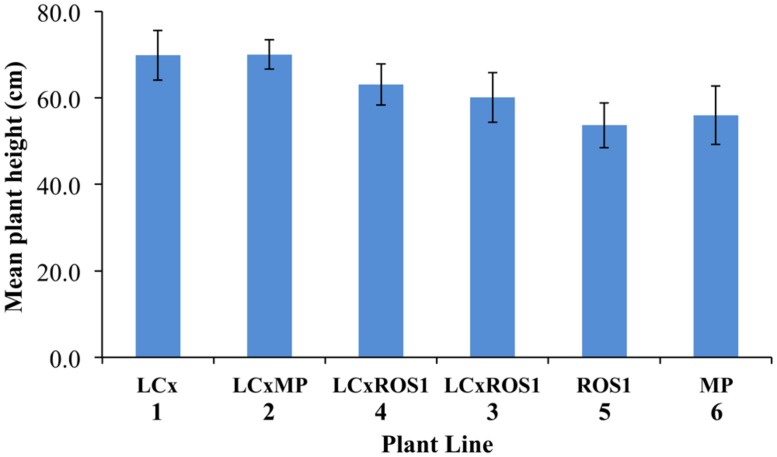
**Average mean plant height of Mitchell petunia lines at the completion of the field trial.** Plant lines are as per **Table 1**. Error bars show the SD values. Collectively, within the *LC* × , *LC* × *MP*, and *LC* × *ROS1* lines, the mean height of ten plants with un-pigmented foliage was 66.2 cm ± 4.87 SD and that of 38 plants with colored foliage 63.55 cm ± 7.04 SD. These means are not statistically different in a *t*-test (*P* = 0.270).

Uniformity of growth was assessed visually prior to field planting. Seedlings in open seed trays grew quicker than seedlings in cell trays, as assessed visually (**Figure 7A**). However, there was no obvious difference between plants of different lines in the field, and there was no significant difference in the mean height of the plants when measured after 3 months of field growth (**Figure 8**).

## DISCUSSION

Introduction of the *35S:ROS1* transgene into either lisianthus or MP resulted in an increase in the anthocyanin production of the transgenic plants, demonstrating that expression of heterologous anthocyanin-related R2R3-MYBs can affect spatial and temporal pigmentation patterns in these species. However, as observed with flavonoid-related R2R3-MYB or bHLH transgenes in other species, the phenotypes resulting varied markedly depending on plant species, organ, developmental age, or environmental conditions. In lisianthus, the *35S:ROS1* transgene induced additional pigmentation in the sepals and petals, but had no apparent affect on anthocyanin biosynthesis in callus or plantlets in culture, mature petals or vegetative parts. Both the spatial and temporal accumulation of anthocyanins was altered in sepals and petals. In petals, precocious pigmentation was induced. This novel pigmentation was confined to the lower half of the petals, while in non-transgenics the first pigmentation to appear is at the petal tips. The fact that most lisianthus cultivars have developmentally late petal pigmentation, with the buds being colorless, is considered a significant disadvantage for their use as cut flowers and ornamentals, and breeding programs have previously tried to introduce an early coloration trait to a wider variety of lisianthus germplasm (Oren-Shamir et al., 1999). The additional pigmentation in sepals was also predominately at the sepal base. A basal pattern of anthocyanin accumulation is common in the floral organs of many species, and in at least some cases is under the control of R2R3-MYB genes (Albert et al., 2011; Cooley et al., 2011). The lack of change in mature petal or vegetative phenotypes was not due to the expression pattern of the *35S:ROS1* transgene, as transcript was detected in a range of flower stages and in leaves (**Figure 2**).

The novel phenotypes observed with the *35S:ROS1* transgene are unlikely to be due to the transformation event itself, as opposed to the action of the *ROS1* gene product. Mitchell petunia is a commonly used line for plant transformation studies, and the changes in phenotype observed here have not been observed when using a wide range of other transformation vectors (e.g., Deroles and Gardner, 1988; Davies et al., 2003; Lewis et al., 2006). The same applies for lisianthus, as phenotypes similar to those found here have not been observed when transgenics of the same cultivars have been generated with either empty vector controls or transformation vectors for various flavonoid biosynthetic genes (Ledger et al., 1997; Schwinn et al., 1997; Deroles et al., 1998; Nielsen et al., 2002).

No information is available on the endogenous regulators of the anthocyanin pathway in lisianthus, but the ability of ROS1 to induce anthocyanin biosynthetic gene expression suggests that, like other species studied to date, R2R3-MYB and bHLH factors control anthocyanin pigmentation in this species. The strong induction of the LBG *ANS*, and the association of this induction with enhanced pigmentation, supports the previous results from gene expression analysis that anthocyanin pigmentation in lisianthus flowers is normally controlled through the regulation of the LBGs (Davies et al., 1993; Oren-Shamir et al., 1999; Noda et al., 2004). The increased transcript levels for the EBG *CHS* in the *35S:ROS1* transgenics shows that *CHS* is also responsive, as a direct or indirect target, to R2R3-MYB factors. The EBGs are commonly principally activated by R2R3-MYB factors that do not require a MBW complex (Feller et al., 2011; Cheynier et al., 2013), however, it is possible they can also be regulated by the anthocyanin-related MBW complex. Based on the color of the precociously pigmented flowers of ROS1-lisianthus, cyanidin-derived anthocyanins accumulated at this stage, not the delphinidin-derived anthocyanins found in the mature petals. This indicates that the genes encoding the flavonoid 3′5′ hydroxylase (F3′5′H) were not up-regulated by the *35S:ROS1* transgene, and matches the pigment types seen in line 54 transgenics containing an antisense *flavonol synthase* transgene that redirects flavonoid biosynthesis from flavonols to anthocyanins (Nielsen et al., 2002). Variability in the activation of F3′5′H genes by R2R3-MYB/bHLH transgenes has been found previously, with ROS1/DELILA but not LC/C1 activating the F3′5′H of tomato (Butelli et al., 2008). Although not effective in lisianthus or tomato, *35S:LC* was able to greatly increased transcript abundance for F3′5′H in MP transgenics (Bradley et al., 1998).

The R2R3-MYBs DPL and PHZ have previously been show to mediate light-activation of anthocyanin biosynthesis in flowers and vegetative tissues of petunia (Albert et al., 2009, 2011). Over-expression of DPL or PHZ in MP resulted in strong anthocyanin pigmentation throughout leaves and stems, without the requirement for high-light levels (Albert et al., 2014). However, this was not the case in the *35S:ROS1* MP. The main novel phenotypes in *35S:ROS1* MP were the surface pigmentation of some callus clumps and an increase in pigmentation of the outer layer(s) of stem tissue. Given that these are the areas exposed to light, it would be interesting to test whether high-light treatment of the transgenics would result in stronger phenotypes. In the case of GMYB10-containing tobacco lines, a significant pigmentation increase only occurred when plants were under high-light conditions (Elomaa et al., 2003). The role of light in regulating anthocyanin production in lisianthus is not known. However, the precocious anthocyanin biosynthesis induced in flowers of *35S:ROS1* lisianthus was much weaker in regions of the petal that were covered by the sepals (**Figure 1**). This indicates that light may promote anthocyanin production in the petals, and that ROS1 over-expression was not able to overcome this effect. Collectively, the limited and very specific changes in pigmentation observed in *35S:ROS1* lisianthus suggest that anthocyanin biosynthesis in this species is under tight temporal and spatial control, and that even in the transgenics the regulation of the pathway remains primarily under the control of endogenous factors rather than being control by ROS1. For both MP and lisianthus, the specificity of pigmentation observed is unlikely to be due to specific expression patterns of the transgene (**Figure 2**).

In MP, *ROS1* induced pigmentation through the regulation of both EBGs (*CHS*) and the LBGs (*ANS*), with a much more marked change seen for *ANS* transcript levels compared to *CHS* (**Figure 5**). This is in line with the function of *ROS1* in antirrhinum, in which it is required for LBG activation and the pigmentation of stem and petal tissue (Schwinn et al., 2006). Although both *CHS* and *ANS* are expressed at significant levels in the petals of the MP transgenics (**Figure 5**) the acyanic petal (due to *an2*) and anther (due to *an4*) phenotypes were not complemented. It was surprising *ROS1* was unable to complement the *an2* and *an4* mutations in MP, since these two loci encode homologous anthocyanin-related R2R3-MYB factors (Quattrocchio et al., 1998, 1999; Schwinn et al., 2006). However, *35S:AN2* expression in MP (W115) also failed to fully complement the *an2* petal phenotype, producing a stem pigmentation phenotype similar to that of *35S:ROS1* but only a weak pigmentation phenotype in petals (Quattrocchio et al., 1999). Neither was full petal pigmentation restored in the DPL and PHZ transgenics (Albert et al., 2011). When the grapevine R2R3-MYBs VvMYBA1, VvMYB5a, and VvMYB5b were over-expressed in MP, VvMYB5b transgenics had some increase in petal anthocyanin production, but only VvMYBA1 showed significant complementation of the *an2* phenotype (Cavallini et al., 2014). In contrast, both the *35S:AN2* and *35S:VvMYBA1* fully complement *an2* in a different genetic background than MP (W115 × W59; Quattrocchio et al., 1999; Cavallini et al., 2014). Ectopic expression of AN2, DPL or PHZ also complemented the phenotype of the *an4* mutation in MP and produced pigmented anthers (Quattrocchio et al., 1998; Albert et al., 2011). Neither over-expression of ROS1 or LC alone resulted in pigmented anthers, but the *35S:ROS1 × 35S:LC* plants did have pigmented anthers, suggesting that alone, ROS1 and LC may not be able to function effectively with all the endogenous regulatory partners but that together they can activate additional biosynthetic gene promoters. Similarly, transient expression of *35S:ROS1* into *35S:LC* MP leaf pieces showed induction of vegetative anthocyanin production (Albert et al., 2009). However, both ROS1 and LC must be able to interact with some endogenous MBW partners, as they do generate some increased pigmentation and analysis of mutant lines of many species has shown the requirement of the full MBW complex for activity.

The results show the large variations produced by both the genetic background of the host and the specific TF transgene used. Often transgenes for single TFs can enhance pigmentation in tissues already producing some pigment, while combinations of TF transgenes may result in *de novo* pigmentation. There is unlikely to be a single explanation to account for the variant patterns produced by the different TF transgenes in different species. It may be related to the availability of adequate precursors, the characteristics of the specific TF introduced, the presence or absence of interacting endogenous TFs, or characteristics of the endogenous TFs. The differences seen for the TF could relate to both varying promoter recognition of specific biosynthetic genes (e.g., F3′5′H, Butelli et al., 2008) and differences in hierarchical regulation of the TFs in different species (Albert et al., 2014). Interestingly, both LC (bHLH) and ROS1 (MYB) can induce pigmentation in leaves of MP (**Figures 4** and **6**), and both in the same sub-epidermal cells, suggesting that simple models based on a specific MYB and/or bHLH being limiting are unlikely to apply. The more recent findings of the involvement of repressor TFs in the MBW complex may help in explaining such results (Yuan et al., 2013; Albert et al., 2014).

The transgenic petunia plant lines generally showed no difference in performance in the greenhouse or field, as measured by seed germination, plant survival or plant stature. The exception was one backcross line of *35S:LC* to MP, which had slightly reduced plant survival in the field. However, as this was not found for any other *35S:LC* lines, including inbreed lines, this is thought to be due to issues with that specific cross or field variation effects. In general, however, no pleiotropic affects such as those found with *35S:VvMYB5* (Mahjoub et al., 2009) were observed from the *35S:LC* or *35S:ROS1* transgenes. There was also no observed gain in plant performance, as measured by the general character of plant stature. It has previously been shown that the *35S:LC* MP plants have increased light saturation and light compensation points (Albert et al., 2009), but this did not have a significant effect under the summer field conditions tested. Replication of the trial at different locations would be desirable to assess whether the lack of difference in overall plant performance between non-transgenic and transgenic lines was consistent under different climatic conditions. The clear phenotypic difference between the plants transferred to the field and those retained in the greenhouse was the induction of anthocyanin pigmentation in the foliage within 4 days of planting. The main environmental differences in the field compared to the greenhouse were higher light (including UV) levels and cooler night time temperatures (as the greenhouse was heated to maintain a minimum temperature of 15∘C). Albert et al. (2009) showed that exposing *35S:LC* MP plants to light levels above those normally found in a greenhouse could strongly promote anthocyanin production in the foliage, so that is likely the main environmental factor responsible for the anthocyanin induction in the field. However, temperatures below 10∘C have also been shown to promote anthocyanin biosynthesis in some species (Gould et al., 2008), so it is possible that the more variable field temperatures could also contribute to increasing pigmentation.

Overall, the results support the potential application of anthocyanin-related TF transgenes for generating novel phenotypes in ornamental crop species. However, the results also highlight the requirement to identify specific TFs or recipient genetic backgrounds that will enable the generation of strong pigment phenotypes, and the need to conduct both greenhouse and field tests of the new plant germplasm.

## Conflict of Interest Statement

The authors declare that the research was conducted in the absence of any commercial or financial relationships that could be construed as a potential conflict of interest.
